# ﻿Morphometric and bioacoustic profiling of *Myotis* cf. *davidii*/*aurascens* (Chiroptera, Vespertilionidae) in East Asia: evidence for a range extension and elevational record

**DOI:** 10.3897/zookeys.1262.151139

**Published:** 2025-12-05

**Authors:** Zhong-Yu Wang, Guang-Hou Chai, Shamshidin Abduriyim

**Affiliations:** 1 College of Life Science, Shihezi University, Shihezi 832003, Xinjiang, China Shihezi University Shihezi China

**Keywords:** *Cytb*, echolocation calls, Pamir Plateau of China, phylogenetic analysis, skull morphology, steppe whiskered bat

## Abstract

During a Chiroptera resource survey conducted in 2023 on the Pamir Plateau of China’s Xinjiang Uygur Autonomous Region, two adult male bat individuals were captured. External morphology examination revealed small-sized individuals characterized by long, narrow ears with straight tragus structures tapering to sharp tips—features diagnostic of the genus *Myotis*. Concurrently, we obtained free-flight echolocation calls from the bats: the signals showed an initial frequency of 65.12 ± 2.12 kHz, terminal frequency of 42.78 ± 1.27 kHz, peak energy frequency of 47.41 ± 1.46 kHz, pulse duration of 2.52 ± 0.26 ms, and inter-pulse interval of 94.01 ± 31.41 ms. Based on skull morphological traits and bioacoustic data, the specimens were preliminarily identified as *Myotis aurascens*. However, our phylogenetic analysis based on the mitochondrial cytochrome *b* (*Cytb*) gene revealed that sequences attributed to *M. aurascens* and *M. davidii* formed a single strongly supported clade, underscoring the unresolved taxonomic status of these species. Irrespective of the exact species assignment, this discovery constitutes the first documented occurrence of the *M. davidii*/*aurascens* complex on the Pamir Plateau, thereby extending the known distribution range in China. Notably, these specimens represent the highest-elevation record for this group to date (3200 m). Our study provides the first comprehensive description of cranial morphology and echolocation signatures for this taxon in a high-altitude habitat. These findings significantly enhance regional biodiversity inventories and offer critical baseline data for plateau ecosystem research.

## ﻿Introduction

The genus *Myotis* represents one of the most speciose groups of bats globally, encompassing over 120 species with remarkable ecological adaptability across diverse habitats (Ruedi et al. 2013). In China, this genus includes 31 recognized species (Yang et al. 2023), many of which exhibit unique biogeographic patterns shaped by the country’s complex topography and climatic gradients (Liu et al. 2023). Among them is the steppe whiskered bat (*Myotis aurascens* (Kuzyakin, 1935)), a species historically confined to the southeastern Mediterranean and Eurasian steppes. Recent findings, however, point to an eastward range expansion into Asia. Previous studies documented its presence in Russia, Mongolia, and the Korean Peninsula (Benda 2004; Tsytsulina et al. 2012; Kim et al. 2015; Bronskov 2017). Until recently, its distribution in China remained largely unknown — Yang et al. (2023) reported the first record in Inner Mongolia, thereby bridging a critical gap between East‐Asian and Central‐Asian populations.

Nevertheless, the taxonomic status of the complex remains unsettled: Yang et al. (2023) concluded, based on a complete mitochondrial genome analysis, that *M. aurascens* and *M. davidii* (Peters, 1869) are distinct species. By contrast, subsequent authors (e.g., Dzeverin 2023) argue that synonymizing these taxa is premature and highlight the need for broader geographic and genetic sampling. In this paper, we adopt a conservative approach: we refer to our specimens as Myotis cf. davidii/*aurascens* to acknowledge that they may belong to either nominal species or a cryptic lineage within this complex.

During a bat-diversity survey conducted on the southern Pamir Plateau of China, two individuals exhibiting diagnostic morphological traits of *Myotis* (including pelage coloration patterns and cranial features) were captured. To validate taxonomic identification, we applied an integrative approach involving molecular phylogenetic analysis (*Cytb*), complemented by craniometric analysis and bioacoustic feature examination. This report presents the first record of the M. cf. davidii/*aurascens* complex on the Pamir Plateau, with emphasis on its elevational distribution and high-altitude habitat occurrence.

## ﻿Material and methods

**Figure 1.**
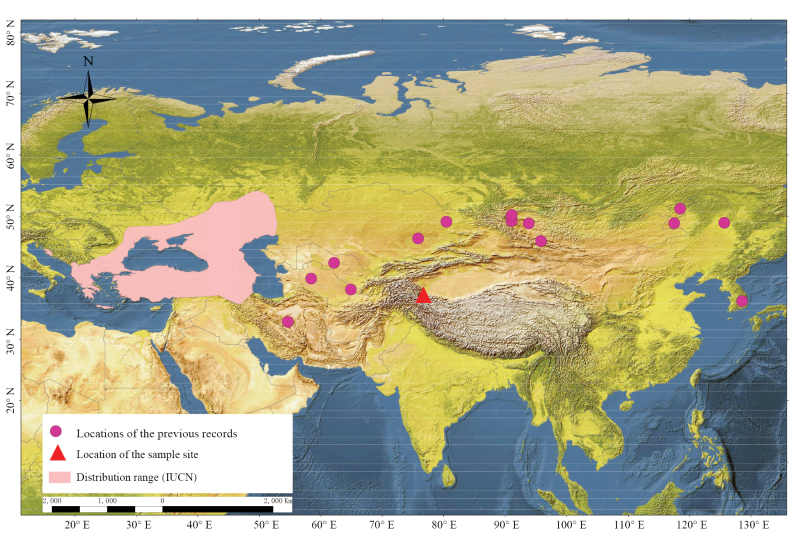
Distribution and sampling locality of M. cf. davidii/*aurascens*. The previously known range is shown in pale pink, with pink circles indicating prior records. The red triangle marks the new sampling site reported in this study, located in southern Xinjiang, China.

During a field survey conducted between July and August 2023 in Taxkorgan Tajik Autonomous County (37°54'24.75"N, 76°47'02.86"E) on China’s southern Pamir Plateau (average elevation ~4500 m a.s.l. in the Xinjiang Uygur Autonomous Region, Fig. 1), we captured two male bats using triple-layered mist nets (Avinet, USA). Concurrently, ultrasonic recording equipment (Song Meter SM4BAT-FS, Wildlife Acoustics, USA) was deployed adjacent to the mist-netting site to systematically acquire echolocation call sequences (Wang and Abduriyim 2025). The detectors recorded continuously in triggered WAV format at a sample rate of 256 kHz, with a minimum trigger frequency of 2 kHz and a minimum recording duration of 1.5 ms.

Age classification (adult, subadult and juvenile) was performed based on the degree of cartilage fusion in the carpal joints (Kunz and Robson 1995). In the laboratory, morphological measurements were taken with a digital caliper (DL91150, Deli Group Co., Ltd., with 0.01 mm precision) following Yang et al. (2023) and Abduriyim et al. (2022), prior to preservation in anhydrous ethanol. The recorded echo waveforms were analyzed using sound analysis software (KALEIDOSCOPE, version 5.4.8) with the FFT size set to 128 and window size to 64 to achieve a 50% overlapping ratio. DNA extraction from wing membrane tissues was performed using the TIANamp Genomic DNA Kit (Tiangen Biotech, Beijing). The mitochondrial cytochrome *b* (*Cytb*) gene’s complete sequence was amplified with mtDNAR3-F and *Cytb*-HH primers (Weyeneth et al. 2008; Puechmaille et al. 2011). The PCR reaction mixture had a total volume of 25 µl, containing 50–150 ng of DNA, 0.4 µmol of each primer, and 12.5 μl of 2×Taq PCR Master Mix (Tiangen, Beijing). The PCR conditions were as follows: an initial denaturation at 94 °C for 7 min, followed by 30 cycles of denaturation at 94 °C for 30 s, annealing at 52 °C for 30 s, extension at 72 °C for 90 s, and a final extension at 72 °C for 5 min. Positive amplicons of the targeted size were confirmed by electrophoresis and sent for bidirectional sequencing to Sangon Biotech (Shanghai, China). Sequences were manually inspected and edited with the SeqMan tool from DNAstar (Librado and Rozas 2009). Following NCBI BLAST analyses, comparable bat *Cytb* gene sequences were downloaded from the NCBI database (Abduriyim et al. 2022). *Cytb* sequences of *M. aurascens* from Benda (2004), Tsytsulina et al. (2012), and Kim et al. (2015) were retrieved for phylogenetic analyses. Two *Cytb* sequences of *Miniopterus schreibersii* (Kuhl, 1817) were included as the outgroup. Phylogenetic trees were constructed using Bayesian-inference (BI) (Ronquist and Huelsenbeck 2003) and maximum-likelihood (ML) (Minh et al. 2020) methods.

## ﻿Results

**Figure 2.**
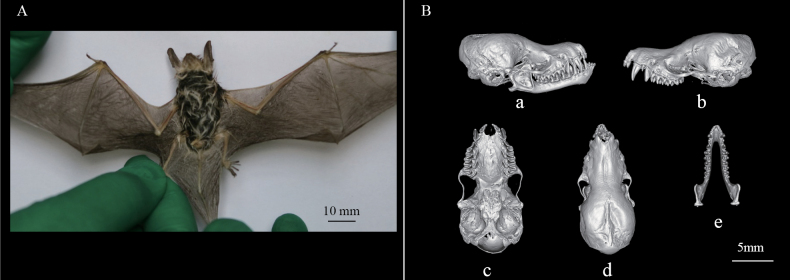
External and cranial morphology of M. cf. davidii/*aurascens*. A. Dorsal view of the bat, showing diagnostic characteristics such as pelage color and ear structure; B. CT scan images of the skull in (a, b) lateral, (c) ventral, and (d) dorsal views, as well as (e) the mandible.

Two adult male bats were discovered in forested areas in the high-valley ridges with abundant vegetation at an elevation of 3206 m above sea level. These two bat specimens exhibited relatively diminutive sizes, indicated by body masses of 4.26 g and 4.58 g. These bats possess long, narrow ears that taper to sharp tips, with a straight and slender tragus. The snout is nearly equal in length to the skull. Their thoracic and dorsal body fur is predominantly ebony, contrasting with white fur on the anterior neck and abdomen. Furthermore, their digits are subtly elongated. The cranial structure displays a flattened, elongated profile, a translucent cranial cavity, narrow eye sockets, and relatively underdeveloped zygomatic arches. The cranial dental formula is 2.1.3.3/3.1.3.3 = 38, with notably elongated upper canines (Fig. 2). These specimens are currently preserved at -80 °C in the College of Life Sciences, Shihezi University (sample IDs TX230805009 and TX230805010).

**Figure 3.**
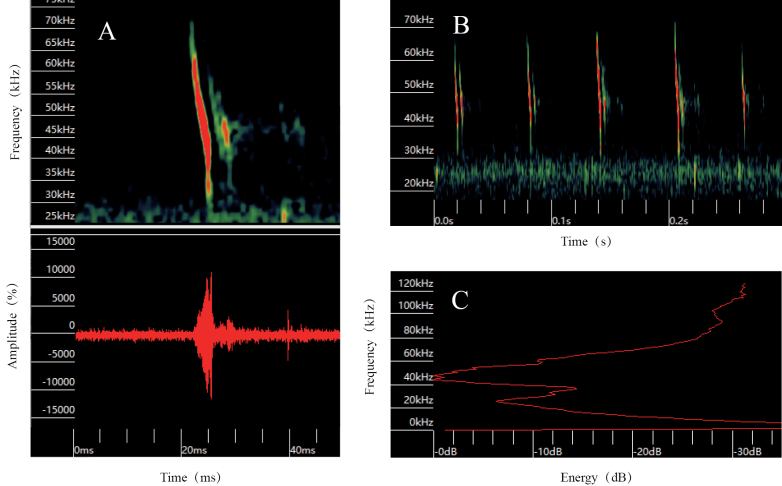
Echolocation call structure of M. cf. davidii/*aurascens* during free flight. A. Spectrogram (above) and waveform (below) over short time scale (ms); B. Spectrogram over long time scale (s) showing pulse interval; C. Energy spectrum of a single cell, showing peak frequency.

Following preliminary screening of 5-minute acoustic data recorded prior to individual capture, analysis of 721 echolocation calls from the resulting 16 audio files revealed that these individuals produce calls with distinctive frequency-modulation characteristics (Fig. 3a, b). In free-flight outdoor conditions, the pulses are composed of a single harmonic. The peak frequency is notably low, with the highest energy peak occurring at 47.41 ± 1.46 kHz (Fig. 3c). The frequency bandwidth is 22.53 ± 3.26 kHz. The pulse duration is relatively short, approximately 2.52 ± 0.26 ms, with an inter-pulse interval of 94.01 ± 31.41 ms (Table 2).

**Figure 4.**
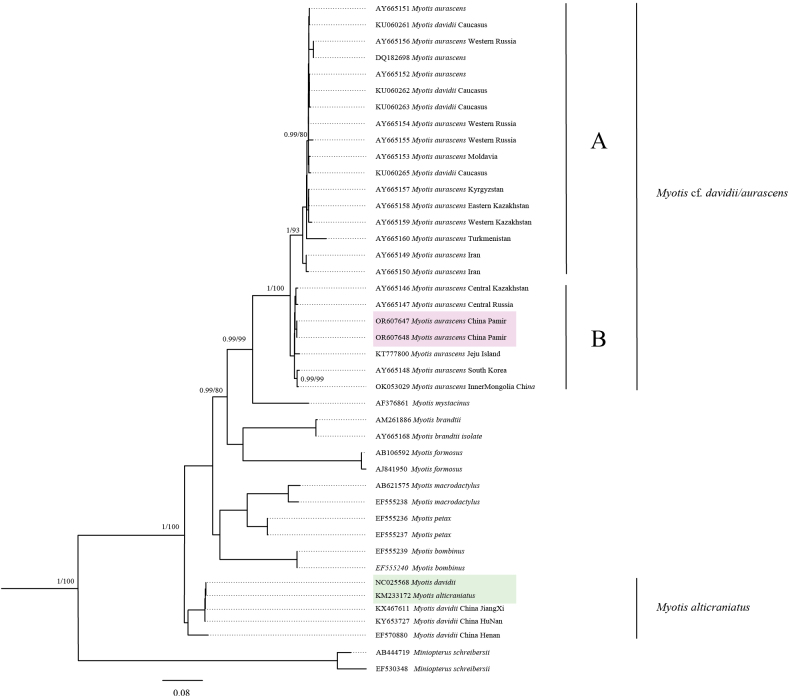
Phylogenetic tree of *Myotis* species based on mitochondrial *Cytb* sequences (1140 bp). The tree was constructed using Bayesian-inference (BI) and maximum-likelihood (ML) methods. *Miniopterus schreibersii* (AB444719 and EF530348) was used as the outgroup. Support values at nodes represent posterior probabilities (BI, ≥0.6) and bootstrap percentage (ML ≥60). The clade containing *M. aurascens* and *M. davidii* is highlighted.

The obtained *Cytb* gene sequences (GenBank accession numbers OR607647 and OR607648) were 1140 bp in length and represented a single haplotype. The GC content was calculated to be 36.78%. A BLAST search revealed a high sequence similarity of 98.78% with *Myotis aurascens*. Phylogenetic analysis showed that our haplotype clustered with other *M. aurascens* and *M. davidii* sequences, forming a single, well-supported clade (M. cf. davidii/*aurascens*) (Fig. 4). Within this clade, two subclades were identified: our sequences grouped with East-Asian individuals (including South Korea). The pairwise genetic distance between the two subclades was 3.4%.

## ﻿Discussion

For a long time, there has been significant taxonomic controversy between *M. davidii* and *M. aurascens* (Dzeverin 2023; Benda et al. 2024). This stems in part from earlier taxonomic identifications based largely on morphological traits alone, and from the fact that many specimens of *aurascens* and *davidii* across museums were poorly documented or mislabeled, thus posing substantial obstacles for subsequent researchers. Based on mitochondrial genome sequences, Yang et al. (2023) concluded that these two species are distinct. However, Ruedi et al. (2021) highlighted numerous Chinese specimens of *Myotis alticraniatus* (Osgood, 1932) that had been misidentified as *M. davidii*, including one complete mitochondrial genome (KM233172; Wang et al. 2016). This particular *M. davidii* mitochondrial genome was utilized by Yang et al. (2023) in their analysis of the phylogenetic relationship between *M. aurascens* and *M. davidii*, which is clearly problematic. In our phylogenetic tree (Fig. 4), which incorporated sequences from their analysis, we obtained results that were consistent with those of Ruedi et al. (2021). This indicates that the conclusions drawn by Yang et al. (2023) regarding *M. aurascens* and *M. davidii* are unreliable.

Our morphological analysis of forearm length, tibia length, and ear length (Table 1) reveals a strong resemblance between the bat specimens collected from the Pamir Plateau and *M. aurascens* populations documented in South Korea, Inner Mongolia, and Mongolia (Benda 2004; Davie et al. 2012; Tsytsulina et al. 2012; Kim et al. 2015; Benda and Paunović 2016; Yang et al. 2023). Consistently, the phylogenetic tree reveals that sequences from these locations and ours were grouped together. However, *M. aurascens* and *M. davidii* sequences formed a singular clade (Fig. 4), making the two species genetically indistinguishable. This contradicts the assertion by Dzeverin (2023) that *M. aurascens* and *M. davidii* are distinct species, while supporting the opposite view of Benda et al. (2012). This underscores the need for further investigation to resolve this taxonomic ambiguity. Therefore, we here refer to our samples as Myotis cf. davidii/*aurascens*.

**Table 1.** External measurements of M. cf. davidii/*aurascens* (Unit: g, mm).

Indices	Xin Jiang, China	Inner Mongolia, China	Mongolia		Jeju Island, Korea
	This study	Yang et al. 2023	Davie et al. 2012		Kim et al. 2015
	*N*=2	*N*=1	♂*N*=54	♀=48	*N*=10
Body Mass	4.26,4.58	6.33	5.5-5.7	5.9-6.1	5.7-7.1
Forelimb Length	35.25,35.29	35.87	33.9-34.3	34.6-35.2	34.71-38.21
Head and Body Length	42.9,36.87	45.1	47.2-48	46.9-47.7	42.6-46.4
Tibia Length	16.58,17.14	15.11	-	-	16.22-18.04
Tail Length	35.33,30.71	32.7	34.7-35.7	35.1-36.1	41.03-45.26
Ear Length	11.12,12.78	13.22	10.3-11.3	10.6-11	13.06-14.98
Ear Width	6.99,9.91	7.25	-	-	-
Tragus Length	6.19,6.34	7.51	6.3-6.5	6.1-6.3	7.64-8.88
Hind Foot Length	7.54,7.88	7.15	-	-	6.42-7.68
Wing Length	105.03,103.93	91.67	-	-	-
Wingspan	226.4,224.84	216.44	-	-	-
Length of the Third Metacarpal Bone	34.04,32.63	28.31	-	-	-
Length of the First Phalanx of the Third Digit	11.23,12.21	10.27	-	-	-
Length of the Second Phalanx of the Third Digit	9.89,10.88	15.55	-	-	-
Length of the Fourth Metacarpal Bone	32,32.09	27.8	-	-	-
Length of the First Phalanx of the Fourth Digit	7.59,8.46	7.85	-	-	-
Length of the Second Phalanx of the Fourth Digit	8.48,9.89	7.49	-	-	-
Length of the Fifth Metacarpal Bone	29.78,30.74	29.55	-	-	-
Length of the First Phalanx of the Fifth Digit	9.6,9.57	16.46	-	-	-

Although we cannot conclusively determine the exact species of our specimens, this finding still represents a new record for the Pamir Plateau—whether as *M. davidii* or *M. aurascens*. This record extends the known distribution range eastward by 700 km from Termez, Uzbekistan, and southward by approximately 1000 km from the area west of Lake Balkhash in Central Kazakhstan, expanding the total distribution area by approximately 700,000 square kilometers.

Intriguingly, our specimens were collected at an elevation of 3206 m — significantly higher than previously documented localities, such as 770 m in Montenegro (Benda 2004). This substantial altitudinal discrepancy highlights the species’ adaptability to high-elevation environments. These findings also imply greater dispersal capacity and thus a broader geographic range for *M. aurascens* than previously recognized (Tsytsulina et al. 2012), potentially reflecting its widespread occurrence across Asia and parts of Europe. However, the observed distribution patterns may also represent fragmented populations rather than a continuous range. Further comprehensive studies are needed to clarify the species’ altitudinal and horizontal distribution limits and to determine whether its populations are interconnected or geographically scattered.

The acoustic profile in this study and that from South Korea (Fukui et al. 2015) exhibit frequency-modulated echolocation call characteristics, including similar peak frequencies, pulse durations, and pulse intervals (Table 2, Fig. 3). However, both the maximum and minimum frequencies in our recordings are lower than those documented in South Korea and Europe (Fukui et al. 2015; Russ 2021), while the bandwidth is comparatively narrower. Variation in echolocation call structure within a species is not uncommon, as geographic divergence, foraging habitats, and behavioral strategies can influence acoustic signatures (Obrist 1995; Obrist et al. 2004).

**Table 2.** Echolocation call features of M. cf. davidii/*aurascens* in free-flight conditions.

Parameters	Range	Mean ± SD
Initial frequency (kHz)	63.00~67.23	65.12 ± 2.12
Terminal frequency (kHz)	41.45~43.98	42.78 ± 1.27
Frequency bandwidth (kHz)	19.27~25.78	22.53 ± 3.26
Peak frequency (kHz)	45.95~48.87	47.41 ± 1.46
Duration time (ms)	2.26~2.77	2.52 ± 0.26
Interval time (ms)	62.60~125.41	94.01 ± 31.41

The observed discrepancies may be attributed to several factors. First, they may reflect adaptive responses to high-altitude environments, given the intermediate geographic position of our sampling site between South Korea and Europe. The extended winter duration at the specimen collection site implies that bats inhabiting the Pamir Plateau must acquire more food within a relatively shorter timeframe to sustain themselves through the prolonged winter. Consequently, they may have undergone adaptive evolution in their echolocation calls to enhance foraging efficiency and success rates (Schnitzler et al. 2003). If true, future genomic studies are needed to elucidate the relationship between echolocation call divergence in Myotis cf. davidii/*aurascens* and its environmental adaptations. Additionally, the capture location was a ridge on the Pamir Plateau with significant ambient water noise, which may drive bats to utilize higher frequency calls for prey detection. Nonetheless, differences in recording equipment and techniques could also contribute to such variations. These explanations remain speculative, as we did not detect this species’ acoustic signals outside this mountainous river basin.

Finally, due to the limited number of specimens collected, our findings cannot fully reflect the local population size and distribution pattern of this species. More in-depth investigations are required to address related ecological questions and draw more comprehensive conclusions.

## ﻿Conclusions

This study reports the first documented occurrence of the M. cf. davidii/*aurascens* complex on the Pamir Plateau. By confirming the presence of this taxon in the region, we address a critical knowledge gap in understanding its distribution across Central Asia. Furthermore, we provide unique echolocation‐call characteristics observed during free-flight behaviour, that deviate from previously recorded patterns attributed to the group. These findings offer foundational insights for future ecological and genetic investigations of M. cf. davidii/*aurascens*. The discovery not only represents a significant new distributional record but also enhances our understanding of biodiversity patterns across multiple dimensions, while presenting new challenges and opportunities for regional conservation practice. Future research should focus on estimating local population size, assessing genetic structure (including nuclear markers), characterising ecological habits and identifying threats faced by this population to support more tailored conservation strategies.

## ﻿Additional information

### Use of AI

No use of AI was reported.

### Author ORCIDs

Zhong-Yu Wang https://orcid.org/0009-0002-6277-942X

Guang-Hou Chai https://orcid.org/0009-0001-2439-1056

Shamshidin Abduriyim https://orcid.org/0000-0002-7038-077X

### Data availability

The obtained *Cytb* gene sequences we obtained have been deposited in the NCBI GenBank databases under accession numbers are OR607647 and OR607648.
